# Upregulation of the pro-apoptotic genes BID and FAS in septic shock patients

**DOI:** 10.1186/cc9181

**Published:** 2010-07-13

**Authors:** Fanny Turrel-Davin, Caroline Guignant, Alain Lepape, Bruno Mougin, Guillaume Monneret, Fabienne Venet

**Affiliations:** 1 Joint Unit Hospices Civils de Lyon - bioMérieux, Hôpital Edouard Herriot, 5 place d'Arsonval, 69437 Lyon cedex 03, France; 2 Hospices Civils de Lyon - Immunology Department, Hôpital Edouard Herriot, 5 place d'Arsonval, 69437 Lyon cedex 03, France; 3 Hospices Civils de Lyon - Intensive Care Unit, Centre Hospitalier Lyon-Sud, Chemin du Grand Revoyet, 69495 Pierre-Bénite, France

## Abstract

**Introduction:**

Lymphocyte apoptosis has been suggested to play a central role in sepsis pathophysiology, and studies in animal models demonstrated that blocking this pathway improves outcome. However, no routine biomarkers of apoptosis are so far available in patients. Thus, the aim of our study was to assess the different biomarkers of apoptosis putatively usable on a routine basis in septic shock.

**Methods:**

Thirteen septic shock patients (sampled twice between days 1 to 2 and days 3 to 5 after diagnosis of shock) and 15 sex-matched and age-matched healthy controls were prospectively enrolled. Apoptosis was measured in lymphocyte subpopulations using flow cytometry (Annexin-V binding, activated caspase-3 and Bcl-2 expressions). Representative pro-apoptotic and anti-apoptotic gene expressions were assessed by quantitative reverse-transcription PCR. Monocyte HLA-DR expression and lymphocyte subpopulation cell counts were measured as markers of sepsis-induced immune dysfunctions. To test for statistical significance, the Mann-Whitney U test was used with correction by the number of tests performed.

**Results:**

Flow cytometric measurements of apoptosis in septic shock patients showed an increased Annexin-V binding on CD4^+ ^T cells and an increased active caspase-3 expression on B cells only at days 3 to 5 (sixfold change and twofold change, respectively). Gene expression analysis showed an increased BCL-XL mRNA and an upregulation of the pro-apoptotic genes BID and FAS in septic shock patients (10-fold change and fivefold change, respectively) compared with healthy controls.

**Conclusions:**

The present study highlights the difficulties encountered in monitoring apoptosis on a routine basis in septic patients, whereas in the same sampling conditions and on the same patients, HLA-DR expression and lymphocyte subpopulation cell counts showed characteristics described in the literature. However, pro-apoptotic genes BID and FAS appear to constitute promising apoptosis markers in our hands.

## Introduction

Despite advances in supportive care, sepsis remains one of the most challenging clinical problems worldwide - constituting the leading cause of death in noncoronary intensive care units (ICUs) [1].

Sepsis initiates a complex immunologic response that varies over time with the concomitant occurrence of both pro-inflammatory and anti-inflammatory mechanisms alternatively predominating [1,2]. After a short pro-inflammatory phase, septic patients enter a stage of protracted immunosuppression illustrated by reactivation of dormant viruses (cytomegalovirus or herpes simplex virus) or infections due to germs normally pathogenic solely in an immunocompromised host [3]. Importantly, the intensity and duration of this phase of immunosuppression appear to be closely correlated with mortality and the development of nosocomial infections [1,4].

Although the mechanistic and molecular bases for sepsis-induced immunosuppression have not yet been fully established, alterations of both innate and adaptive immune responses have been described [1,4]. In particular, an increased leukocyte apoptosis has been observed in septic patients [5]. Two major apoptotic pathways are activated: the extrinsic or death receptor-initiated caspase-8 mediated pathway, involving the superfamily of TNF receptor members; and the intrinsic or mitochondria-initiated caspase-9 pathway, which interplays between pro-apoptotic and anti-apoptotic members of the BCL-2 family [6].

Interestingly, numerous studies in animal models of sepsis showed that blocking programmed cell death improves outcome after septic challenge [6]. This approach could thus represent a potential innovative therapeutic strategy in septic shock. Since no clinical signs of apoptosis have been described, however, there is thus an urgent need to develop robust biomarkers available on a routine basis for the monitoring of this phenomenon. In patients, Weber and colleagues recently observed an extensive apoptosis of circulating lymphocytes after severe sepsis (upregulation of BIM and BID gene expression, and a downregulation of the anti-apoptotic molecules BCL-2 and BCL-XL) [7].

Parallel to this work, the aim of the current study was to test the capacity of such well-known markers of apoptosis (Annexin-V binding, active caspase-3 and BCL-2 expressions measured by flow cytometry, and the balance between pro-apoptotic and anti-apoptotic mRNA expressions measured by quantitative reverse-transcription (qRT) PCR to detect the activation of this cell-death pathway in circulating blood of septic shock patients in the condition of routine laboratory monitoring. Despite increased mRNA expressions of the pro-apoptotic genes BID and FAS, our results highlight the difficulties encountered in the monitoring on a routine basis of apoptosis in septic patients.

## Materials and methods

### Patients and controls

The present study was conducted in 13 consecutive patients with septic shock prospectively included according to the diagnostic criteria of the American College of Chest Physicians/Society of Critical Care Medicine [8]. Patients were admitted to the two participating ICUs (one medical, one surgical) of the Lyon-Sud University Hospital (Hospices Civils de Lyon, France). Septic shock was defined by an identifiable site of infection, by hypotension persisting despite fluid resuscitation and requiring vasopressor therapy, and by evidence of a systemic inflammatory response manifested by at least two of the following criteria: temperature >38°C or <36°C, heart rate >90 beats/minute, respiratory rate >20 breaths/minute, and white blood cell count >12,000/mm^3 ^or <4,000/mm^3^. The onset of septic shock was defined by the beginning of vasopressive therapy. The only exclusion criteria were patients younger than 18 years old and the absence of circulating leukocytes. Patients were treated according to the standardized recommendations of our ICUs. Severity at the onset of shock was assessed by the Simplified Acute Physiology Score II (range: 0 to 194) [9].

Laboratory analyses were performed on residual ethylenediamine tetraacetic acid-anticoagulated blood after completing routine follow-up performed in the ICUs, in accordance with the guidelines for clinical research of our institute (which, in this case, waived the need for informed consent). Arterial blood samples were obtained at days 1 to 2 (D1-2) and days 3 to 5 (D3-5) after the onset of shock (one sample per period). After blood sampling in the ICUs, tubes were transported at 4°C to the immunology laboratory within 2 hours in accordance with our usual routine protocol for monocyte HLA-DR monitoring [10]. Flow cytometry staining was first performed as described below, and the remaining blood was then processed to isolate peripheral blood mononuclear cells (PBMCs) by Ficoll density gradient centrifugation (within 3 hours). To provide panels of control values for mRNA quantification and flow cytometry analysis, 15 sex-matched and age-matched healthy individuals (age 60.6 ± 5.37 years; eight females, seven males) with no known co-morbidities were also included.

### Cell isolation before mRNA analysis

PBMCs were isolated within 3 hours after blood collection by Ficoll-Paque density gradient centrifugation (Amersham Biosciences, Björkgatan, Sweden) and washed with PBS, while the remaining red blood cells were lysed. Cellular concentration was determined and cell viability was assessed using the trypan blue exclusion method before the cell pellet was conserved in RNA Cell Protect (Qiagen, Hilden, Germany) at -20°C until RNA extraction.

### RNA extraction and cDNA synthesis by reverse transcription

Total RNA was extracted from PBMCs using RNeasy Plus Mini kits (Qiagen). For each RNA extraction, the residual genomic DNA was digested using the gDNA Eliminator spin column (Qiagen) and RNA was diluted in 30 μl elution buffer. RNA quantity was determined for each sample using a Qubit (Invitrogen, Carlsbad, CA, USA) according to the manufacturer's instructions. cDNA was then synthesized from 20 ng total RNA using the WT-Ovation™ System (NuGEN, San Carlos, CA, USA) powered by Ribo-SPIA™ technology. Briefly, first-strand cDNA is generated using a unique first-strand DNA/RNA chimeric primer mix and reverse transcriptase. mRNA was subsequently fragmented, allowing the synthesis of a second strand by DNA polymerase. Finally, the Ribo-SPIA™ amplification provides highly efficient amplification of DNA sequences.

### Quantitative PCR analysis of apoptotic genes and HLA-DR

Messenger RNA expression was quantified using quantitative PCR. Briefly, PCR reactions were performed in a LightCycler^® ^480 instrument using the associated SYBR Green I Master Mix according to the manufacturer's instructions (Roche Molecular Biochemicals, Indianapolis, IN, USA). For amplification, the reaction volume was 20 μl and the cycling conditions were an initial denaturation step of 95°C for 5 minutes (one cycle), followed by 45 cycles of a touch-down PCR protocol (20 seconds at 95°C, 15 seconds annealing at 68 to 58°C and 15 seconds extension at 72°C), a melting curve of 95°C for 1 second, 60°C for 10 seconds and 95°C for 5 minutes, and, to finish, a cooling cycle at 40°C for 30 seconds.

The mRNA expression of the housekeeping gene peptidylpropyl isomerase B (PPIB) encoding for cyclophilin B was investigated using specific cDNA standards and ready-to-use primer mixes obtained from Search-LC (Heidelberg, Germany). The efficiency of PPIB mRNA levels has been previously demonstrated as the reference for target mRNA quantification in human peripheral blood [11]. The apoptotic genes (BCL-2, the anti-apoptotic forms of BCL-XL, BAX, BAK, BIM, BID and FAS) and HLA-DRA PCR amplicons were obtained with the primer combinations presented in Table 1. Serial dilutions of the cDNA were prepared in quadruplicate to generate standard curves. Relative standard curves, describing the PCR efficiency of apoptotic genes and PPIB, were created and used to perform efficiency-corrected quantification with the LightCycler^® ^software version 1.5. An internal calibrator was used to compare each cDNA amplification. The results are expressed as the normalized ratio of BCL-2, BCL-XL, BAX, BAK, BIM, BID FAS and HLA-DRA mRNA relative to PPIB mRNA. The qRT-PCR results were only included in the analysis if PPIB mRNA values were comprised within the standard curve - which accounts for most of the missing values.

**Table 1:** Quantitative reverse-transcription PCR performance, parameters and primers

Gene symbol	UniGene	**Efficiency**^**a**^	**Error**^**b**^	**Primer sequences**^**c**^
*BCL2*	Hs.150749	1.87	0.0059	5'-GCGACGACTTCTCCCGC-3'
				5'-GCGATGTTGTCCACCAGG-3'
*BCL-XL*	Hs.516966	1.89	0.0101	5'-GTAGTGAATGAACTCTTCCG-3'
				5'-GTATCCCAGCCGCCGTTCTC-3'
*BAX*	Hs. 624291	1.75	0.0297	5'-CAAACTGGTGCTCAAGG-3'
				5'-CCAACCACCCTGGTCTTGG-3'
*BAK*	Hs.485139	1.91	0.0175	5'-GCCACCAGCCTGTTTGAG-3'
				5'-CTGCCACCCAGCCACCC-3'
*BIM*	Hs.469658	1.95	0.0112	5'-GAGCCACAAGACAGGAGC-3'
				5'-CCATTGCACTGAGATAGTGG-3'
*BID*	Hs.591054	1.69	0.0184	5'-TGGTGTTTGGCTTCCTCCAA-3'
				5'-GAATCTGCCTCTATTCTTCCC-3'
*FAS*	Hs.244139	1.93	0.0198	5'-GGAATCATCAAGGAATGCAC-3'
				5'-CCAAACAATTAGTGGAATTGG-3'
*HLA-DRA*	Hs.520048	2.04	0.0195	5'-GCCAACCTGGAAATCATGACA-3'
				5'-AGGGCTGTTCGTGAGCACA-3'

^a^Amplification efficiency of standard curve. ^b^The standard curve error value. ^c^Top sequence is forward primer, bottom is reverse primer.

### Apoptosis measurements by flow cytometry

One hundred microliters of whole blood were incubated with PE-Cy7-labeled anti-CD3 or anti-CD19 antibodies and PE-Cy5-labeled anti-CD4 or anti-CD8 antibodies (BD Pharmingen, San Jose, CA, USA), and were then lysed using VersaLyse lysing solution (Beckman-Coulter, Hialeah, FL, USA).

Apoptosis induction in each specific lymphocyte subpopulation - CD4^+ ^T cells (CD4^+^CD3^+^), CD8^+ ^T cells (CD8^+^CD3^+^) and B cells (CD19^+^) - was assessed using Annexin-V binding, intracellular active caspase-3 and Bcl-2 expression measurements.

Regarding Annexin-V binding experiments, according to the manufacturer's protocol, lysed samples were incubated for 15 minutes with PE-labeled Annexin-V (Annexin-V-PE apoptosis detection kit; BD Pharmingen) and measured on a flow cytometer within 30 minutes (FC500; Beckman-Coulter). Results are expressed as percentages of respective cell populations positive for Annexin-V binding. A threshold for positivity was set up based on nonstained controls.

For active caspase-3 and Bcl-2 intracellular stainings, following two washes, lysed cells were permeabilized using Cytofix/Cytoperm reagent (BD Pharmingen) and were incubated with either PE-labeled anti-active caspase 3 antibodies or PE-labeled anti-Bcl-2 antibodies (BD Pharmingen). Isotype control antibodies were used to determine nonspecific binding. After one further wash, cells were analyzed by flow cytometry. Results are expressed as the mean fluorescence intensity.

### Circulating lymphocyte phenotyping and monocyte HLA-DR measurements by flow cytometry

**Figure 1:**
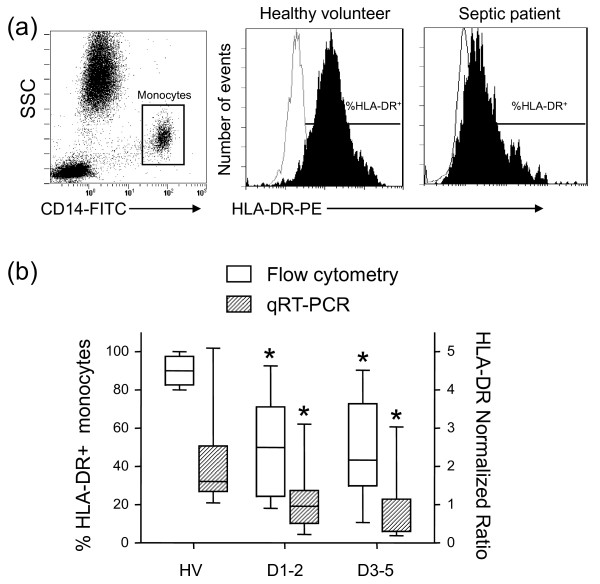
**HLA-DR expression in septic shock patients and healthy controls**. **(a) **Cell surface expression of HLA-DR measured by flow cytometry. A representative CD14 versus side-scattered light (SSC) dotplot and representative HLA-DR linear histograms gated on monocytes in one healthy volunteer and one septic patient (white histogram = isotype control). **(b) **Boxplot representations of mHLA-DR measurements by flow cytometry and quantitative reverse transcription (qRT)-PCR (white boxes, percentage of HLA-DR-expressing monocytes; dashed boxes, normalized ratio of HLA-DRA mRNA level relative to the housekeeping gene peptidylpropyl isomerase B) measured in whole blood or mononuclear cells from septic shock patients or healthy controls (*n *= 10). Values were measured at days 1 to 2 (D1-2, *n *= 7) and days 3 to 5 (D3-5, *n *= 10) for septic patients. Normal values: >90% for flow cytometry and 2 ± 0.3 for mRNA expression. Comparison versus healthy volunteers' (HV) values (means) performed using the Mann-Whitney U test with correction for the number of tests performed: *P < 0.025. mHLA-DR values expressed as the number of bound antibodies per cell (AB/c) were 15,985 ± 2,331 at D1-2 and 11,057 ± 2,691 at D3-5 in patients (>15,000 AB/c as indicative of immunocompetence [28]).

Monocyte HLA-DR expression was assessed as described previously [10]. Representative examples of mHLA-DR staining by flow cytometry in one patient and one volunteer are presented in Figure 1a. The following lymphocyte subsets were analyzed: total T lymphocytes (CD45^+^CD3^+^), CD4^+ ^T lymphocytes (CD45^+^CD4^+^CD3^+^), and CD8^+ ^T lymphocytes (CD45^+^CD8^+^CD3^+^). Monoclonal antibodies were used according to the manufacturer's recommendations: FITC-labeled anti-CD45, PC5-labeled anti-CD3, PE-labeled anti-CD4, and ECD-labeled anti-CD8 (Beckman-coulter). The red cells were lysed on the automated TQ Prep system (Beckman-Coulter). The absolute count was performed using Flow-count fluorospheres according to the manufacturer's recommendations (Beckman-Coulter). Results are expressed as numbers of lymphocytes per microliter of blood.

### Statistical analysis

Values are expressed as medians and interquartile ranges (Q1 to Q3). Comparisons between healthy controls and septic shock patients (at D1-2 or D3-5) were made using the nonparametric Mann-Whitney U test, with *P *< 0.025 considered statistically significant with correction by the number of tests performed.

## Results

### Characteristics of the septic patient cohort

Thirteen patients with septic shock (six women and seven men) and 15 healthy volunteers (sex-matched and age-matched) were enrolled in the present study. The demographic and clinical characteristics of the cohort are presented in Table 2. None of these patients were immunocompromised (HIV, cancer, immunosuppressive treatments). Seven patients received adjunctive corticosteroid treatment during their ICU stay.

**Table 2:** Demographic and clinical data for septic shock patients

Parameter	Patients (*n *= 13)
Age at admission (years)	60 (4)
Gender	
Male	7
MacCabe score	
0	10
1	3
Main diagnosis category	
Medical	8
Surgical	5
SAPS II score at diagnosis of shock	51 (3)
Adjunctive corticosteroid treatment	7
Delay shock - ICU (days)	≤1
Infection diagnosis	
Radiologically diagnosed	1
Surgically diagnosed	0
Microbiologically documented	11
Bacilli Gram-negative	5
Bacilli Gram-positive	6
Fungi	1
Others	1
Type of infection	
Community-acquired	10
Hospital-acquired	3
ICU-acquired	0
Site of infection	
Pulmonary	6
Abdominal	4
Others	3
Mortality	
Survivors	10

Data presented as *n *or mean (standard error of the mean). ICU, intensive care unit; SAPS II, Simplified Acute Physiology Score II.

The total number of leukocytes was increased in septic patients at D1-2 and D3-5 compared with healthy volunteers (Mann-Whitney U test; Table 3). In contrast, the total lymphocyte cell count was significantly diminished at D1-2 in shock patients compared with normal values. Regarding circulating lymphocyte subpopulations, a global decrease in T cells (CD4^+ ^and CD8^+^) was observed in septic shock patients at D1-2 (Table 3). Our patient cohort was also characterized by a severely reduced percentage of HLA-DR-expressing monocytes measured by flow cytometry and reduced HLA-DRA mRNA expression measured by qRT-PCR in comparison with normal values at D1-2 and at D3-5 (Figure 1b).

**Table 3:** Circulating blood leukocyte phenotyping in septic shock patients

	Healthy controls	Septic shock patients	
		
		Days 1 to 2	Days 3 to 5
Leukocytes	5658 (4,954 to 6,146)	14,673 (9,562 to 18,446)	15,390* (10,097 to 25,235)
Lymphocytes	1549 (1,295 to 1,831)	701* (432 to 1,531)	1,110 (634 to 1,482)
T cells	1090 (902 to 1,470)	421* (209 to 547)	529 (335 to 1,031)
CD4^+ ^T lymphocytes	890 (569 to 1,141)	220* (115 to 354)	479 (218 to 827)
CD8^+ ^T lymphocytes	320 (228 to 349)	155* (36 to 207)	158 (70 to 304)

Results expressed as the median (interquartile range, Q1 to Q3) number of cells/μl. Blood samples were obtained from 13 septic shock patients at days 1 to 2 and at days 3 to 5 after diagnosis of shock. Circulating leukocyte immunophenotyping was systematically performed by flow cytometry. The following leukocyte subsets were analyzed: total number of leukocytes, total number of lymphocytes, T cells (CD45^+^CD3^+^), and CD4^+ ^(CD45^+^CD4^+^CD3^+^) and CD8^+ ^(CD45^+^CD8^+^CD3^+^) T-lymphocyte subpopulations. Comparison versus healthy volunteers values (mean) performed using the Mann-Whitney U test with correction for the number of tests performed: *P < 0.025.

### Annexin-V binding, caspase-3 and Bcl-2 expressions measured by flow cytometry

**Figure 2:**
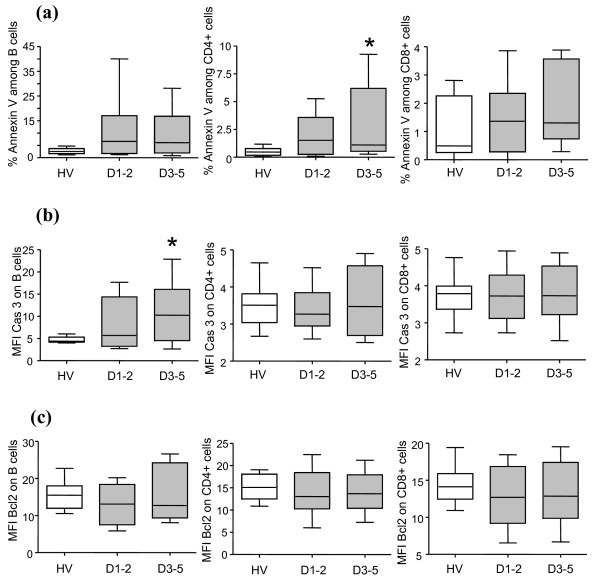
**Flow cytometric measurement of Annexin-V binding, active caspase-3 and Bcl-2 expressions in lymphocyte subpopulations**. **(a) **Percentage of cells binding Annexin-V, and the mean fluorescence intensities (MFIs) of **(b) **active caspase-3 and **(c) **Bcl-2 expressions were measured by flow cytometry in CD19^+^, CD4^+ ^and CD8^+ ^T-lymphocyte subpopulations of healthy volunteers (HV) (open boxes, *n *= 12) and septic shock patients (grey boxes) at days 1 to 2 (D1-2, *n *= 11) and days 3 to 5 (D3-5, *n *= 10). Data presented as boxplots. Comparison versus healthy volunteers values (means) performed using the Mann-Whitney U test with correction for the number of tests performed: *P < 0.025.

We further assessed apoptosis by flow cytometry (Annexin-V binding, active caspase-3 and BCL-2 expressions). We observed a trend toward an increased Annexin-V binding on CD4^+ ^T cells and CD19^+ ^B cells at D1-2 but no change on CD8^+ ^T cells (Figure 2a). This increase became significant at D3-5 on CD4^+ ^T cells only.

Active caspase-3 expression, the central caspase in the apoptosis pathway [12], was increased in CD19^+ ^cells of septic patients at D3-5 compared with healthy volunteers, but no significant changes were observed in CD4^+ ^and CD8^+ ^T cells (Figure 2b).

Regarding BCL-2, whatever the time point and T-cell subpopulation investigated, its protein level measured by flow cytometry remained similar to that of healthy controls (Figure 2c).

### Pro-apoptotic and anti-apoptotic gene mRNA expressions measured by qRT-PCR

**Figure 3:**
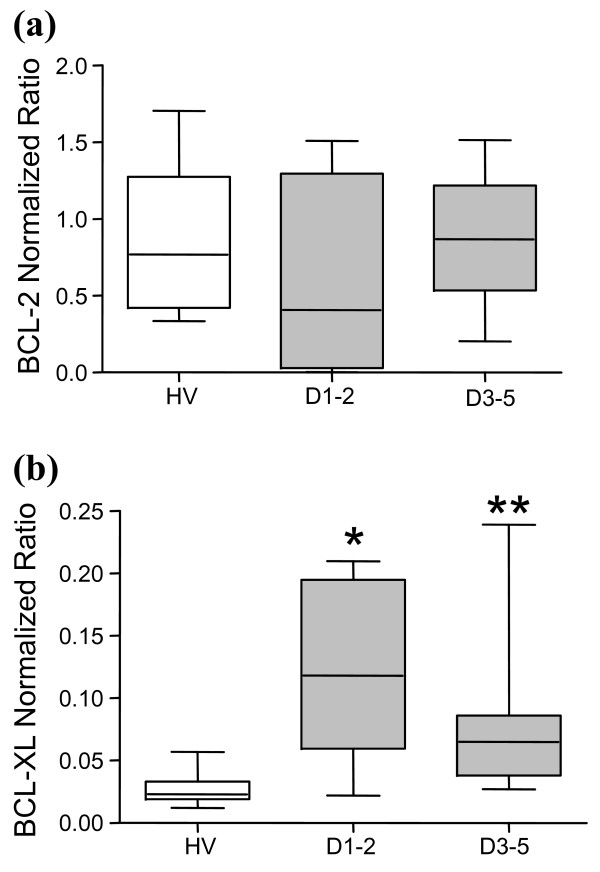
**Gene expression of anti-apoptotic Bcl-2 family members measured by quantitative reverse-transcription PCR**. mRNA expressions of anti-apoptotic genes **(a) **BCL-2 and **(b) **BCL-XL were quantified by quantitative reverse-transcription PCR in healthy controls (open boxes, *n *= 12) and septic shock patients (grey boxes) at days 1 to 2 (D1-2, *n *= 6) and days 3 to 5 (D3-5, *n *= 8) after diagnosis of shock. The targeted mRNA levels were normalized to that of the housekeeping gene peptidylpropyl isomerase B and are thus expressed as normalized ratio. Data presented as boxplots. Comparison versus healthy volunteers' (HV) values (mean) performed using the Mann-Whitney U test (*P < 0.025, ** *P *< 0.005).

We then analyzed mRNA expressions of the anti-apoptotic genes BCL-2 and BCL-XL in septic shock patients and controls (Figure 3). BCL-2 mRNA expression was slightly decreased in septic shock patients at D1-2 compared with healthy individuals but returned to normal values at D3-5. Surprisingly, BCL-XL mRNA levels were significantly increased in shock patients at both time points compared with healthy volunteers.

**Figure 4:**
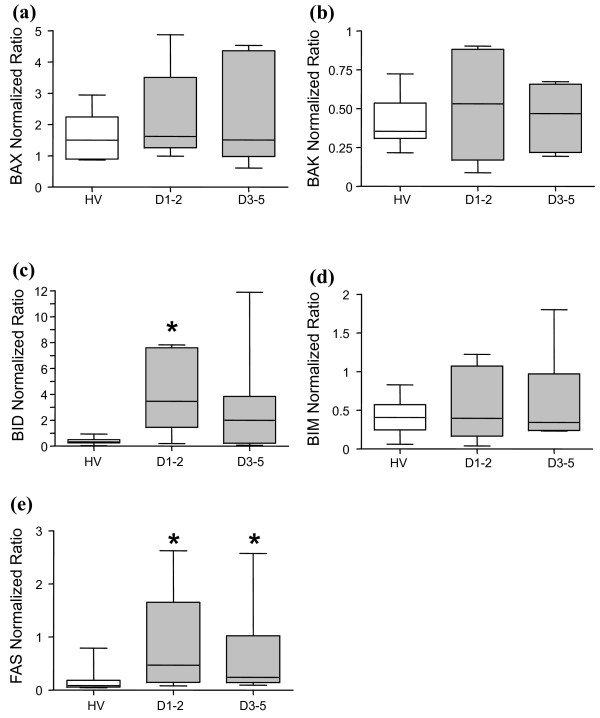
**Gene expression of pro-apoptotic Bcl-2 family members and death receptor FAS measured by qRT-PCR**. mRNA expressions of pro-apoptotic genes **(a) **BAX, **(b) **BAK, **(c) **BID, **(d) **BIM and **(e) **FAS were quantified by quantitative reverse-transcription PCR in healthy volunteers (HV) (open boxes, *n *= 11) and septic shock patients (grey boxes) at days 1 to 2 (D1-2, *n *= 6) and days 3 to 5 (D3-5, *n *= 8). The targeted genes mRNA levels were normalized to that of the housekeeping gene peptidylpropyl isomerase B. Data presented as boxplots. Comparison versus healthy volunteers values (mean) performed using the Mann-Whitney U test (*P < 0.025).

We then measured gene expressions for the BAX-like proteins (BAX and BAK), the BH3-only proteins (BID and BIM) and the extrinsic receptor FAS in septic shock patients and healthy volunteers (Figure 4). Only a trend toward increased gene expressions of BAX, BAK and BIM was observed in patients compared with controls (Figure 4a, b, d). A significantly increased BID mRNA expression was measured in patients at D1-2 in comparison with healthy volunteers (Figure 4c).

Concerning FAS mRNA expression, we observed a significant increase in septic patients at D1-2 and D3-5 compared with healthy controls (Figure 4e).

## Discussion

Despite studies investigating the role of apoptosis in septic patients and showing a marked increase in circulating lymphocyte apoptosis associated with a profound and persistent lymphopenia and with poor outcome [1,13-15], none have measured programmed-cell death in routine laboratory conditions. The present study was thereby designed to test the capacity of well-known markers of apoptosis to detect this phenomenon on a routine basis in a cohort of septic shock patients.

Despite the small size of this cohort, clinical characteristics were similar to those described in the literature for septic shock patients. Moreover, patients were characterized by a decrease in circulating lymphocyte number and a dramatic downregulation of monocyte HLA-DR expression at the protein and mRNA levels in accordance with the literature [10,16].

Regarding apoptosis measurement, flow cytometry results showed an increased apoptosis at D3-5 in septic shock patients in CD4^+ ^T cells (as indicated by Annexin-V binding) and in B cells (as indicated by active caspase 3 staining). These results, however, were not confirmed by the measurement of a decreased BCL-2 expression. In parallel, mRNA expression underlined a surprisingly gradual increase of the BCL-XL gene level until D3-5 compared with healthy controls, a trend toward an increased gene expression of BAX, BAK and BIM, coupled with a massive upregulation of BID and FAS pro-apoptotic genes. Of note, however, no difference in apoptosis markers was observed between patients receiving or not receiving adjunctive corticosteroid treatment (data not shown).

Contrary to other studies measuring apoptosis in septic patients, no clear-cut increase in Annexin-V binding was observed on B cells and CD8^+ ^T cells, whereas total lymphocyte and CD4^+ ^and CD8^+ ^lymphocyte numbers were decreased in these studies as well as in our study [14,15]. Moreover, no significant decrease in BCL-2 gene expression was observed in our hands. One explanation for these discrepancies may be linked with the difficulties encountered in the monitoring, on a routine basis, of apoptosis in circulating blood of septic shock patients. In particular, the delay between blood drawing in ICU and tube processing in the laboratory (2 hours) may have lead to sample deterioration, and could thus explain the slight difference between our results and the literature. Indeed, such problems have been described in a recent study investigating the potential of the measurement of Annexin-V binding on lymphocytes as a biomarker in emergency departments [17].

Regarding mRNA experiments, gene-expression patterns were examined in specific cellular subsets (PBMCs) in the present study rather than in peripheral whole blood cells (total mRNA extracted using the PAXgene™ Blood RNA system (PreAnalytix, Hilden, Germany)) as performed in the study by Weber and colleagues [7]. The initial goal was to avoid possible interference with neutrophils, which are known - contrary to lymphocytes - to present with decreased apoptosis in critically ill patients [18,19]. The second goal was to work with only one blood sample for flow cytometry and qRT-PCR, thereby reducing the volume of blood collected from patients. The counterpart, however, is that the process of PBMC purification increases the delay between blood sampling and mRNA extraction (3 hours), and thus might have further increased sample deterioration. This is indeed illustrated in the present study by the high number of missing values in qRT-PCR experiments due to the low mRNA extraction yield. Another drawback may be that PBMCs still constitute a mixture of several cell subpopulations (monocytes and various lymphocyte subsets). As these subpopulations could have different apoptotic responses after sepsis, this could limit the value of the direct comparison between qRT-PCR and flow cytometry results. If we are to develop a biomarker of apoptosis usable on a routine basis, however, single cell-population purification is not possible (time and blood consuming). PAXgene™ tubes may thus appear the appropriate technique for mRNA study in human blood although they similarly present limitations, including mixed cell populations and an overabundance of globin gene expression.

With that said, in the current study the expressions of the pro-apoptotic genes BID and FAS were markedly upregulated in septic shock patients. This upregulation suggests that, as opposed to the markers listed before, BID and FAS might be robust biomarkers of apoptosis in routine sampling conditions. In Weber and colleagues' article, a similar induction of BID gene expression was observed in early severe septic patients compared with critically ill patients [7]. Moreover, in experimental models of sepsis, BID knockout mice showed nearly complete protection from sepsis-induced lymphocyte apoptosis and a marked survival advantage in polymicrobial sepsis [20,21].

Regarding FAS, an increased expression was observed on leukocytes, particularly on neutrophils and monocytes, of humans treated with endotoxin [22]. In septic patients, PBMCs also exhibited an increase in FAS and FAS ligand expressions, in correlation with mortality [23]. Moreover, studies have shown that inhibiting the FAS-mediated apoptotic pathway (FAS-ligand-deficient mice or administration of a FAS fusion protein) reduced mortality in an experimental model of sepsis [20,24,25]. Similarly, FAS inhibition by siRNA given 30 minutes after cecal ligation and puncture improved survival by up to 50% and reduced apoptosis and organ damages in both the liver and the spleen [26].

## Conclusions

Lymphocyte depletion, in part due to apoptosis, has been suggested to play an essential role in the pathophysiology of sepsis [5,6], and numerous studies suggest that targeting apoptosis could thus represent a potential innovative therapeutic strategy in septic shock [5,6,27]. A step necessary before the use of such therapy in septic patients, however, is to develop biomarkers of apoptosis available on a routine basis to identify patients who could benefit from this treatment before its initiation (that is, patient stratification). Our results underline the difficulties encountered in the monitoring of apoptosis in septic shock patients in routine laboratory conditions, whereas the usual markers of immunoparalysis, such as decreased monocyte HLA-DR expression, are easily detected (both at protein and mRNA levels) in the same sampling conditions. This observation suggests it is crucial to develop specific robust and reliable tools for apoptosis monitoring in septic patients to ensure clinical sample quality in routine laboratory conditions. Among the markers tested in the present study, BID and FAS mRNA expressions appear promising candidates. This deserves to be confirmed in a larger and ideally multicentric clinical study.

## Key messages

• Routine monitoring of apoptosis in septic shock patients deserves specific protocols.

• Septic patients present with decreased monocyte HLA-DR expression and lymphopenia.

• Among parameters measured in this article, FAS and BID mRNA appear to be promising apoptosis markers in septic shock.

## Abbreviations

BAK: BCL2 antagonist killer; BAX: BCL2-associated × protein; BCL2: B-cell lymphoma 2; BCL-XL: B-cell lymphoma - extra large; BID: BH3-interacting domain death agonist; BIM: BCL2-interacting protein BIM; D1-2: days 1 to 2; D3-5: days 3 to 5; HLA: human leukocyte antigen; ICU: intensive care unit; PBMC: peripheral blood mononuclear cell; PBS: phosphate-buffered saline; PCR: polymerase chain reaction; PPIB: peptidylpropyl isomerase B; qRT: quantitative reverse transcription; TNF: tumor necrosis factor.

## Competing interests

The authors declare that they have no competing interests.

## Authors' contributions

FT-D and FV conceived the study, participated in its design and data analysis, and drafted the manuscript. FV and CG established the flow cytometric methodology, and participated in data acquisition and analysis. AL was involved in clinical sample and data acquisition. FT-D established the PCR methodology and the analysis, and designed the primers. GM participated in the study design. FV, GM and BM revised the manuscript for intellectual content. All authors have read and approved the final manuscript.
